# Utilizing Natural Language Processing of Narrative Feedback to Develop a Predictive Model of Pre-Clerkship Performance: Lessons Learned

**DOI:** 10.5334/pme.40

**Published:** 2023-05-03

**Authors:** Christina Maimone, Brigid M. Dolan, Marianne M. Green, Sandra M. Sanguino, Patricia M. Garcia, Celia Laird O’Brien

**Affiliations:** 1 Associate director of research data services, Northwestern IT Research Computing Services, Northwestern University, Evanston, Illinois, USA; 2 Associate professor of medicine and medical education and director of assessment, Northwestern University Feinberg School of Medicine, Chicago, Illinois, USA; 3 Raymond H. Curry, MD Professor of Medical Education, professor of medicine, and vice dean for education, Northwestern University Feinberg School of Medicine, Chicago, Illinois, USA; 4 Associate professor of pediatrics and senior associate dean of medical education, Northwestern University Feinberg School of Medicine, Chicago, Illinois, USA; 5 Professor of obstetrics and gynecology and medical education, Northwestern University Feinberg School of Medicine, Chicago, Illinois, USA; 6 Assistant professor of medical education and assistant dean of program evaluation and accreditation, Northwestern University Feinberg School of Medicine, Chicago, Illinois, USA

## Abstract

**Background:**

Natural language processing is a promising technique that can be used to create efficiencies in the review of narrative feedback to learners. The Feinberg School of Medicine has implemented formal review of pre-clerkship narrative feedback since 2014 through its portfolio assessment system but this process requires considerable time and effort. This article describes how natural language processing was used to build a predictive model of pre-clerkship student performance that can be utilized to assist competency committee reviews.

**Approach:**

The authors took an iterative and inductive approach to the analysis, which allowed them to identify characteristics of narrative feedback that are both predictive of performance and useful to faculty reviewers. Words and phrases were manually grouped into topics that represented concepts illustrating student performance. Topics were reviewed by experienced reviewers, tested for consistency across time, and checked to ensure they did not demonstrate bias.

**Outcomes:**

Sixteen topic groups of words and phrases were found to be predictive of performance. The best-fitting model used a combination of topic groups, word counts, and categorical ratings. The model had an AUC value of 0.92 on the training data and 0.88 on the test data.

**Reflection:**

A thoughtful, careful approach to using natural language processing was essential. Given the idiosyncrasies of narrative feedback in medical education, standard natural language processing packages were not adequate for predicting student outcomes. Rather, employing qualitative techniques including repeated member checking and iterative revision resulted in a useful and salient predictive model.

## Background and need for innovation

Medical educators have only recently begun to explore how machine learning tools such as natural language processing (NLP) can be leveraged to create efficiencies in the review of textual data. As clinical competency committees (CCC) become more prevalent, educators have sought better ways to organize and aggregate narrative data used to make judgements about learner performance [1,2]. NLP has been used to estimate CCC ratings [3] and detect residents with performance difficulties [4]. It has also been used to identify words and phrases associated with feedback on specific competency areas [5] and entrustment levels [6]. In undergraduate medical education, NLP has identified characteristics of feedback associated with entrustment ratings [7] and found differences in words used to describe clerkship students by race and under-represented status [8]. However, the above findings may not be applicable to narrative feedback to medical students in the pre-clerkship setting, nor to a comprehensive system that includes feedback from multiple assessments, settings, and sources. The purpose of this paper is to describe the processes and lessons learned when applying NLP to develop a predictive model of pre-clerkship medical student performance as part of a longitudinal, comprehensive assessment system.

The Northwestern University Feinberg School of Medicine has implemented faculty competency committee review of pre-clerkship narrative feedback since 2014 through its portfolio assessment system [9,10]. Each learner portfolio includes all student assessment data across multiple courses, including faculty and peer assessments of small group work, clinical performance assessments, observed structured clinical examinations (OSCEs), and direct observations of clinical skills. Assessments include narrative feedback and categorical ratings such as “Below expectations,” “Meets expectations,” and “Exceeds expectations.”

A summative competency committee review of each student portfolio occurs at the end of the 20-month-long pre-clerkship curriculum. Each portfolio is read by trained faculty clinicians who assess five competency domains: patient care, communication, professionalism, teamwork, and self-regulated learning. Reviewers are trained to detect patterns in narrative feedback reflecting behaviors that reappear over time and judge whether this behavior will affect future clinical performance. Reviewers are asked whether they feel the student is consistently meeting the benchmarks of each competency domain and can respond: a) Yes; b) Almost; or c) Not Yet. Approximately 20% of students each year receive an “Almost” or “Not Yet” rating in at least one competency and participate in additional skills practice before progressing to the clerkship phase. Regardless of ratings, all students receive narrative feedback from the committee on their strengths and areas for growth.

Our previous research has shown that competency committee review of portfolio data detects concerning behaviors in students that might otherwise go unnoticed and that these behaviors can impact future clerkship performance (e.g., patterns of repetitive lateness) [10]. Furthermore, our reviewers and students report that narrative comments provide the highest quality information about behaviors that cannot be communicated through categorical ratings alone. These findings are consistent with prior research on the value of narrative feedback [11,12].

While narrative comments are a critical component of our assessment process, the time and effort required to read and assess narrative data are substantial. Committee reviewers report that it takes approximately 90 minutes to review and provide feedback for each student’s portfolio; given approximately 165 students per class and that each portfolio is reviewed by at least two faculty, this represents a considerable investment of human resources.

In 2019, we began to explore how NLP could improve our existing assessment system, acknowledging that the goal was to support, not replace, faculty judgement. There are limitations to automated assessment of complex behaviors, particular in medical education. Feedback given to learners is often nuanced and hedging in nature [13], and the purpose and context of an assessment is critical to correctly interpreting feedback [14]. It is imperative to proactively plan for these challenges when constructing any algorithm to extract patterns and themes in narrative feedback.

## Goal of Innovation

In this paper, we describe how we employed NLP to analyze narrative feedback and build a predictive model of pre-clerkship medical student performance in a comprehensive competency-based assessment system. In the sections that follow, we discuss the approach taken and lessons learned when applying NLP in this setting.

This study was reviewed and approved by the Northwestern University Institutional Review Board (STU00210653).

## Steps taken for Development and Implementation of Innovation

Social scientists have argued that an iterative, sequential, and inductive approach to computational text analysis will yield more defined and interpretable concepts of interest than a standard deductive approach [15]. We applied this framework to our NLP analysis with the goal of identifying features, or characteristics, of text that were interpretable and intuitive to both faculty and learners.

The NLP analysis was conducted by a specialist from the research computing department of our parent university (C.M.) using R programming language (version 4.1.0, Vienna, Austria). Over two years, C.M. met multiple times with the co-authors on this paper to learn about the pre-clerkship curriculum and assessments at Feinberg and to gain an understanding of the context in which this feedback was provided to students. This allowed her to engage more deeply in an initial, exploratory analysis of the narrative data to generate features associated with student performance. Below, we describe the steps we took used to create a useful and salient predictive model using NLP.

### Processing and cleaning the data

Portfolio assessment data from 2014–2019 were combined to identify relevant features of the narrative feedback. Data from 2020 and 2021 were reserved to evaluate the model. The training dataset included 910 students who went through the pre-clerkship portfolio review between 2014 and 2019; 314 students were included in the test data from 2020–2021. Data were cleaned to remove irrelevant comments (e.g., “N/A”). On average, each student received 318 narrative comments suitable for inclusion in the analysis, with comments having an average length of 196 characters (approximately 30 words). We created a dichotomous outcome variable by classifying students into two groups: those meeting all competency benchmarks (students “Ready” to progress) and those who received “Almost” or “Not Yet” (students “Not Yet Ready”).

### Feature generation

We initially applied several approaches that are commonly described in the literature. One common NLP technique involves counting how many times individual words and short phrases appear in a body of text. However, in our dataset this technique resulted in detecting words and phrases that, although significantly associated with performance outcomes, lacked practical meaning and were not indicative of performance. Examples of such words include “wry” and “product.” Moreover, we did not find clear “red flag” terms that would immediately signal a student was “Not Yet Ready.” These findings suggested that this technique was insufficient to generate a set of features that would be trusted by and helpful to faculty reviewers.

A second approach involved the use of existing tools. In many available open-source NLP packages, there are well-established tools that estimate the positive or negative sentiment of text [16]. However, these tools may not transfer to medical education where a word such as “good,” a positive indicator in most settings, often indicates below-average performance when applied to learners in medical education. Similarly, words like “nauseous” are generally negative in common data sets, but when used by a clinical preceptor are often describing the content of a specific student presentation. Likewise, techniques such as Latent Dirichlet Allocation (LDA) [17] can automatically group related words together into what are known as “topics,” but these approaches were more useful for identifying clusters of similar comments than for identifying features predictive of student outcomes.

Given the limitations of these available tools, we manually grouped words and terms that were individually predictive into custom-built topics. This process was inductive and iterative. Groups included words that were functionally similar (e.g., positive or negative adjectives) or addressed an aspect of performance frequently commented on by portfolio reviewers (e.g., presentation skills). Topics were also constructed according to the source of the feedback, particularly for peers and standardized patients. For example, if the word “uncomfortable” is used in feedback from faculty, it usually refers to the student. However, if “uncomfortable” is used in feedback from a standardized patient, it usually refers to the patient’s own feelings. While both uses may be indicative of an area where a student may need additional practice, they point to different issues.

The proposed topics were presented to three co-authors (B.D., M.G., S.S.) for member checking, all of whom have experience reviewing portfolios and who provided feedback on the interpretability of the groups. The topics were iteratively revised to meet two goals: relevance for faculty reviewers and contribution to performance of a predictive model. The process was similar to axial coding in qualitative methodology [18].

### Feature testing and model building

Topics were tested for consistency over time, and whether they were similarly predictive across demographic sub-groups of students, including gender and racial/ethnic identity. Inconsistent topics or topics that raised concern for bias were removed from the model. For example, if a topic or feature predicted a positive outcome for students identifying as male but a negative outcome for students identifying as female, then this topic was revised or removed from the predictive model given concern for bias within that feature.

In addition to topics, other variables were tested for inclusion in the model. This included categorial ratings from assessment forms and overall word counts. Because the assessment system and content of the portfolios changed throughout the years this data was collected, all variables were normalized per year to adjust for variations. Several predictive models were tested, including logistic regression, XGBoost, support vector machines, naïve Bayes, and elastic net logit.

## Outcomes of Innovation

The best-fitting model used a combination of both categorical ratings and narrative feedback for maximum accuracy. Three types of features were included in the model:

1. Sixteen topic groups of words and phrases, which are displayed in Table 1.
2. Number of words in a student’s portfolio (relative to other students from the same year). Students who are “Not Yet Ready” averaged more words than their peers.
3. Below expectations ratings per competency area: The number of “below expectations” categorical ratings that a student received on pre-clerkship assessment forms in each of the five competency domains.

**Table 1:** Description of topics predictive of pre-clerkship performance at the Feinberg School of Medicine.


TOPIC NAME	DESCRIPTION	EXAMPLE WORDS AND TERMS	HAVING MORE OF THESE TERMS INCREASES THE LIKELIHOOD OF A STUDENT BEING:

**Change**	Phrases that indicate the student may need to change a behavior	“I would encourage,” “make sure,” “I suggest,” “be more”	Not Yet Ready

**Common Negative Terms**	Frequently appearing words	although, but, little, more, not	Not Yet Ready

**Frequency Words**	Words that indicate the frequency with which something occurred	few, generally, instance, many times, mostly, multiple times, occasionally, occasions, once, rarely, seldom, several times, sometimes, tend, tendency, usually	Not Yet Ready

**Hedging**	Words that soften a comment or phrase, or add uncertainty to a comment	at least, fairly, less, little bit, maybe, might, much, nearly, perhaps, possibly, rather, seem, slightly, sometimes seemed, somewhat, sort of, while	Not Yet Ready

**Late/Absent**	Words and phrases suggesting a potential issue with student attendance or timeliness	absence, absences, absent, arrive on time, attendance, be on time, late	Not Yet Ready

**Negative Adjectives**	Adjectives associated with negative or problematic behaviors	argumentative, arrogant, cautious, detached, detrimental, disengaged, disinterested, disorganized, flustered, irrelevant, rambling, reticent, superficial, underprepared, vague, withdrawn	Not Yet Ready

**Negative Standardized Patient Feelings**	Negative feeling words appearing in feedback from standardized patients	confused, disconnected, fearful, frustrated, nervous, overwhelmed, uncomfortable, uneasy, unsure, upset	Not Yet Ready

**Negative Terms**	Words and phrases associated with negative or problematic behaviors	allow others, ask more questions, error, excuses, fail, forget, impression, interrupt, looking down, more consistent, more detail, nervous energy, not paying attention, omit, phone, reminder, repetition, skip, struggle, suffer, surprise, trouble, unable, unfortunately, wrong	Not Yet Ready

**Speak-up**	Phrases that indicate the student needs to participate more in group conversations or speak up	speak up, participate more, hear more, contribute more, more vocal	Not Yet Ready

**Common Positive Terms**	Frequently appearing, positive words	active, clear, clearly, effective, excellent, great, helpful, leader, leadership, professional, respectful, team member, thoughtful, well organized	Ready

**Positive Adjectives**	Positive general adjectives (not specific skills or attributes)	amazing, awesome, brightest, exceptional, exemplary, extraordinary, fantastic, highly, impressive, incredible, indispensable, instrumental, integral, outstanding, remarkable, star, superb, superior, superlative, talented, terrific, tremendous, unparalleled, wonderful	Ready

**Positive Adverbs**	Positive adverbs	absolutely, actively, consistently, effectively, effortlessly, excellently, extremely, extremely well, incredibly, professionally, wonderfully	Ready

**Positive Attributes**	Positive personal attributes	asset, calm, caring, cheerful, clearly prepared, compassionate, consistently prepared, eloquent, empathic, engaging, excellent communicator, friendly, great attitude, hardworking, incredibly helpful, insightful, kind, proactive, reassuring, valuable, very effective, warm, welcoming	Ready

**Positive Presentation**	Words and phrases suggesting good presentation skills	concise presentations, effective presentations, engaging presentation, excellent presentation, interactive, organized presentations, strong presentation, useful information, well presented	Ready

**Positive Skills**	Words and phrases indicating desired behaviors and skills	bedside, calm demeanor, clarity, clear understanding, consistently demonstrated, create, decision making, encouraged others, enhance, excellent communication, excellent eye contact, excellent rapport, facilitate, good explanation, good eye contact, good questions, good technique, great teacher, implement, insightful question, often volunteered, open ended questions, probing questions, same page, skilled, took initiative, utilize, well articulated, well written	Ready

**Positive Teamwork**	Words and phrases suggesting good teamwork skills	active member, awesome group, enthusiastic member, excellent group, excellent team, great teammate, group focused, integral member, strong member, team dynamic, teamwork, teamwork skills	Ready


We chose logistic regression as the model type for the predictive analysis. While other model types had similar performance, logistic regression had the advantage of being easier to compute, less prone to overfitting, and more familiar to competency committee faculty. Logistic regression also allows for uncertainty estimates (standard errors) on predicted values. The model had an area under the curve (AUC) value of 0.92 on the training data and 0.88 on the test data from 2020–2021.

Table 2 illustrates predicted competency committee review outcomes for three example students in the 2020–2021 test dataset. Each student received an overall model score ranging from 0 (likely ready) to 1 (likely not yet ready). The characteristics of the narrative feedback impacting this prediction are also described, along with the actual review outcome for comparison. The examples demonstrate that while the model can correctly predict an overall outcome of a human review, the topic groups themselves are very general. Human review is still necessary to provide students with specific and actionable feedback in particular competency domains.

**Table 2:** Use cases of example students comparing model predictions of the test dataset to actual review outcomes.


	STUDENT “A”	STUDENT “B”	STUDENT “C”

**Predicted outcome**	Overall score of 0.001, indicating this student is highly likely to be found “Ready.”	Overall score of 0.27, indicating this student may be at risk of being found “Not Yet Ready.”	Overall score of 0.88, indicating this student is highly likely to be found “Not Yet Ready.”

**Characteristics of narrative feedback impacting the prediction**	Relatively higher number of words and terms in the “Positive Attributes,” “Positive Adjectives,” and “Positive Skills” topics. Relatively lower number of words and terms in the “Change,” “Hedging,” and “Late/Absent” topics.	Relatively higher number of words and terms in the “Speak Up,” “Hedging,” and “Negative Adjectives” topics. Relatively higher than average number of words and terms in the “Positive attributes,” and “Positive Presentation” topics. Relatively lower number of words and terms in “Positive Teamwork” and “Positive Skills” topics.	Relatively higher number of words and terms in the “Late/Absent,” “Change,” and “Frequency Words” topics. Relatively lower number of words and terms in the “Positive Teamwork,” “Positive Attributes,” and “Positive Adverbs” topics.

**Actual competency committee review outcome**	Student is considered “Ready” across all competency domains. Specifically exceeds expectations in Patient Care domain.	Student is considered “Not Ready” in the Teamwork domain. Student is directed to meet with faculty mentor to improve ability to contribute to group discussions.	Student is considered “Not Ready” in the Professionalism and Teamwork domains. Student is directed to work with an educational support team to grow effective leadership skills, and to work with a faculty mentor to improve accountability.


## Reflection

This study presents the process and results of applying NLP techniques to predict student performance in a comprehensive pre-clerkship assessment system. Narrative feedback provides the most robust information about student performance but reviewing comments in a timely manner is challenging. We found that the best-performing predictive model included manually constructed topic groups, the total word count of narrative within each portfolio, and the number of below-expectation ratings. This model has allowed us to provide our competency committee faculty with visual summaries of each student’s narrative feedback and to create an early detection system to facilitate offering support to students prior to the summative review. These projects will be discussed in future work.

Given the complexity of analyzing narrative data from numerous assessments, sources, and settings, we found an iterative, inductive approach to model development was critical; methods others have found successful such as LDA [5] did not successfully predict medical student outcomes. Traditional NLP methods are often developed using large text data sets such as product reviews, news articles, or online encyclopedia entries. However, as others have described in non-NLP work related to medical education, the text in narrative feedback has unique characteristics [19]; we caution others against relying on standard open-source NLP packages without close examination of the results. The meaning of words used in feedback depends entirely on the context.

Institutional context is also important. We expect that our approach to creating features and building a predictive model would work well at other medical schools that systematically collect large amounts of narrative feedback to pre-clerkship learners, and we believe many of the concepts underlying the topics to be widely relevant and generalizable. For example, the “Hedging” topic is similar to a theme found in Ginsburg et al.’s [13] qualitative analysis on learner feedback, and “Positive Attributes” and “Positive Skills” are similar to concepts found in the work of Rojek et al. [8]. However, the specific words that comprise a topic are likely to differ, given how assessment contexts vary across institutional settings.

It is also important to monitor how models created from historical data will be applicable to future cohorts. The assessment system, competency standards, and curriculum at Feinberg have evolved since 2014. Assignments and assessments have been added, dropped, or moved to a different time point in the curriculum. To address this, we aggregated words and phrases to create features that generalize across time and do not focus too narrowly on the experience of just a few students. We also looked for features that show a consistent relationship with student outcomes from year to year. As the system continues to evolve over time, it will be critical to review existing measures to ensure their continued relevance.

Finally, and perhaps most importantly, it is critical to ensure that any features or models of narrative feedback are equitable. As we built the model, we ensured that a given feature was indicative of the same outcome for students of different genders and race/ethnicity groups. While statistical models cannot eliminate all issues that arise from data generated by humans about other humans, we can closely monitor the performance and impact of such models and choose methods that align with the values and mission of the school.

There is still much to learn about how NLP can enhance performance assessment of our learners. Our experiences applying NLP to narrative feedback in the pre-clerkship setting have shown promising early results.

## Data Accessibility Statement

Due to the difficulty of de-identifying narrative feedback, the data used in this project is not openly available.
